# Insecticide exposure alters feeding and impairs ovary development in a solitary bee

**DOI:** 10.1007/s10646-025-03019-y

**Published:** 2026-02-18

**Authors:** Clara Stuligross

**Affiliations:** 1 Biology Department, Kalamazoo College, Kalamazoo, MI USA; 2 Department of Entomology and Nematology, University of California, Davis, Davis, CA USA

**Keywords:** Pesticide, Neonicotinoid, Pollinator, Carryover effect, Ovary, Feeding

## Abstract

Insecticide exposure is a growing concern due to its implication in population declines and disruption of key ecosystem services such as pollination. Bees and other beneficial insects can be exposed to insecticides multiple times throughout their lives and across generations, with impacts on behavior and reproduction. To investigate mechanisms underlying reduced reproduction in bees exposed to insecticide over time, I quantified the direct and carryover effects of insecticide exposure on the feeding and ovary development of the solitary bee, *Osmia lignaria*. Bees were exposed to the insecticide, imidacloprid, as larvae and/or as adults in a fully crossed design over two years. Insecticide exposure to bees as adults, but not as larvae, reduced pollen and nectar feeding and impaired ovary maturation. These findings reveal mechanisms underlying insecticide effects on bees, indicating that negative impacts on reproduction may be attributed to reduced ovary development driven by the anti-feeding effects of imidacloprid, even at low levels of exposure.

**Supplementary Information:**

The online version contains supplementary material available at 10.1007/s10646-025-03019-y.

## Introduction

Widespread declines of beneficial insects threaten critical ecosystem services, including the pollination of crops and wild plants (Klein et al. 2007; Ollerton et al. 2011; Wagner et al. 2021). These declines result from multiple independent and interacting factors (Goulson et al. 2015; Wagner et al. 2021); among them are a set of new or greatly accentuated stressors created by the intensive use of synthetic chemicals like pesticides (Goulson et al. 2015; Woodcock et al. 2016; Sánchez-Bayo and Wyckhuys 2019; Wagner et al. 2021; Wan et al. 2025). Pesticide effects are particularly concerning in agricultural contexts where insect pollinators contribute over $150 billion annually in pollination services (Gallai et al. 2009). The toxicity of applied pesticides has increased dramatically over the last 25 years, with neonicotinoid insecticides in particular driving the highest toxic exposures for pollinators like bees (Whitehorn et al. 2012; Rundlöf et al. 2015; DiBartolomeis et al. 2019; Douglas et al. 2020; Schulz et al. 2021).

Given the ever-increasing extent of human-dominated land use such as intensive agriculture, bees may encounter pesticides repeatedly or consistently throughout their lives and over multiple generations. Adult bees can be exposed during foraging and nesting via contact with pesticides in the air, on plants, on nest materials, or by consuming contaminated pollen and nectar. Immature bee larvae can similarly be exposed via food or by contact with contaminated nest materials, such as soil or leaves, throughout their development (Sgolastra et al. 2019; Willis Chan et al. 2019; Raine and Rundlöf 2024). How exposure across the life cycle impacts reproduction is known only for a few species (Rundlöf et al. 2015; Stuligross and Williams 2021), and whether effects carry over to impact subsequent generations is largely untested (Willis Chan and Raine 2021; Stuligross and Williams 2021). Both immediate and carryover effects have the potential to substantially impact population dynamics over time (Woodcock et al. 2016; Stuligross and Williams 2021). Understanding the long-term impacts of pesticide exposure is critical to inform risk assessment and ensure persistence of bee populations across landscapes.

Insecticides can delay nesting and egg-laying by exposed bees (Wu-Smart and Spivak 2018; Leza et al. 2018; Stuligross and Williams 2020), which may reduce reproduction. This may be especially true for solitary bees that nest over short time periods—often only a few weeks per year—and have relatively low fecundity (Bosch and Vicens 2006; Stuligross and Williams 2020). Such delays suggest a potential impact of insecticides on reproductive physiology or behavior, as adult females must mature eggs within their ovaries before laying them. Indeed, exposure of adult bees to insecticides is associated with impaired ovary development in some studies (Baron et al. 2017; Sgolastra et al. 2018), but not others (Azpiazu et al. 2019; Siviter et al. 2020). Because these results are not consistent among bee species nor the insecticide class studied, further investigation of insecticide impacts on ovary development is important to reveal the mechanisms underlying reduced reproduction in exposed bees. One straightforward possibility is that pesticides could impact ovary development indirectly through their well-documented impacts on feeding and foraging motivation (Gill and Raine 2014; Lämsä et al. 2018; Muth et al. 2020; Stuligross et al. 2023). However, despite evidence that diet is important for ovary development (Hoover et al. 2006; Cane 2016; Tanaka et al. 2019), we have little understanding of the relationship between insecticide exposure, feeding, and ovary development (Baron et al. 2017).

Ovary development is an essential stage of the reproductive process, and successful, continuous oocyte maturation is required to maximize reproduction throughout an adult bee’s life (Danforth et al. 2019). In addition, rapid oocyte maturation at the onset of adult emergence appears to be beneficial, allowing solitary bees to take full advantage of their reproductive lifespans (Stuligross and Williams 2020). In the solitary bee genus *Osmia*, oocytes begin to develop slowly during pre- and overwintering, although full maturation only occurs during early adulthood (Wasielewski et al. 2011; Lee et al. 2015; Sgolastra et al. 2016; Cane 2016). Therefore, insecticide exposure during either early development (e.g. larvae) or early adulthood could impact ovary development. Additionally, exposure could affect larvae and adults differently due to life stage-specific physiological differences such as detoxification mechanisms, avoidance capacity, and changing expression or sensitivity of insecticide binding sites during development (Dupuis et al. 2012; Grünewald and Siefert 2019). Despite such potential for important and unique effects on larvae, the carryover effects of larval insecticide exposure on ovary development have not been explored.

Together, these gaps highlight a need for studies that consider both the direct effects of insecticides on ovary maturation and the possibility that exposure may alter ovary development indirectly through changes in feeding. Evaluating these pathways across different life stages can reveal how both current and past exposure history impacts reproductive outcomes.

I investigated the direct and carryover effects of sublethal insecticide exposure on the feeding (nectar and pollen) and ovary development of the solitary blue orchard bee, *Osmia lignaria*. I used the neonicotinoid insecticide imidacloprid, a widely-used systemic insecticide that is toxic to bees (Jeschke et al. 2011; Goulson 2013; Craddock et al. 2019). Bees can be exposed to imidacloprid from treated crops as well as untreated wild plants due to the environmental movement of neonicotinoids (Botías et al. 2015; Rundlöf et al. 2022).

To test how current and past insecticide exposure influence feeding and ovary development, I conducted a laboratory experiment with a crossed exposure design. Adult bees were exposed to insecticide at high or low concentrations, or unexposed, and were sourced from two past insecticide exposure backgrounds (with or without larval exposure; Stuligross and Williams 2021). This experimental paradigm provided combinations of bees exposed or not exposed to insecticide over two years. I measured the nectar consumption, pollen consumption, and ovary maturation. I expected insecticide exposure to reduce feeding and impair ovary development, but that the carryover effects would differ in magnitude, with adult exposure having greater impacts than the carryover effects from past larval exposure.

## Methods

### Study system and bee origin

The blue orchard bee *Osmia lignaria* is a solitary univoltine species native to North America. It is a model species for solitary bee biology and is also widely used for orchard pollination (Torchio 1985; Bosch and Kemp 1999). Individual bees for this experiment were sourced from two past insecticide exposure backgrounds. In the previous year, I conducted an experiment in outdoor field cages; half of the cages were treated with the neonicotinoid insecticide imidacloprid at the label rate (AdmirePro^®^, Bayer Crop Science; 10.5 oz/acre; 767 ml/ha), and *O. lignaria* flying within the cages provisioned offspring in nests (Stuligross and Williams 2020). I used those offspring for this study—so bees either had a past history of no insecticide exposure, or they were exposed to insecticide as developing larvae via the pollen and nectar provisioned by their mothers (Stuligross and Williams 2021). Details of larval imidacloprid exposure and residue analyses are reported in Stuligross and Williams (2020).

**Fig. 1:**
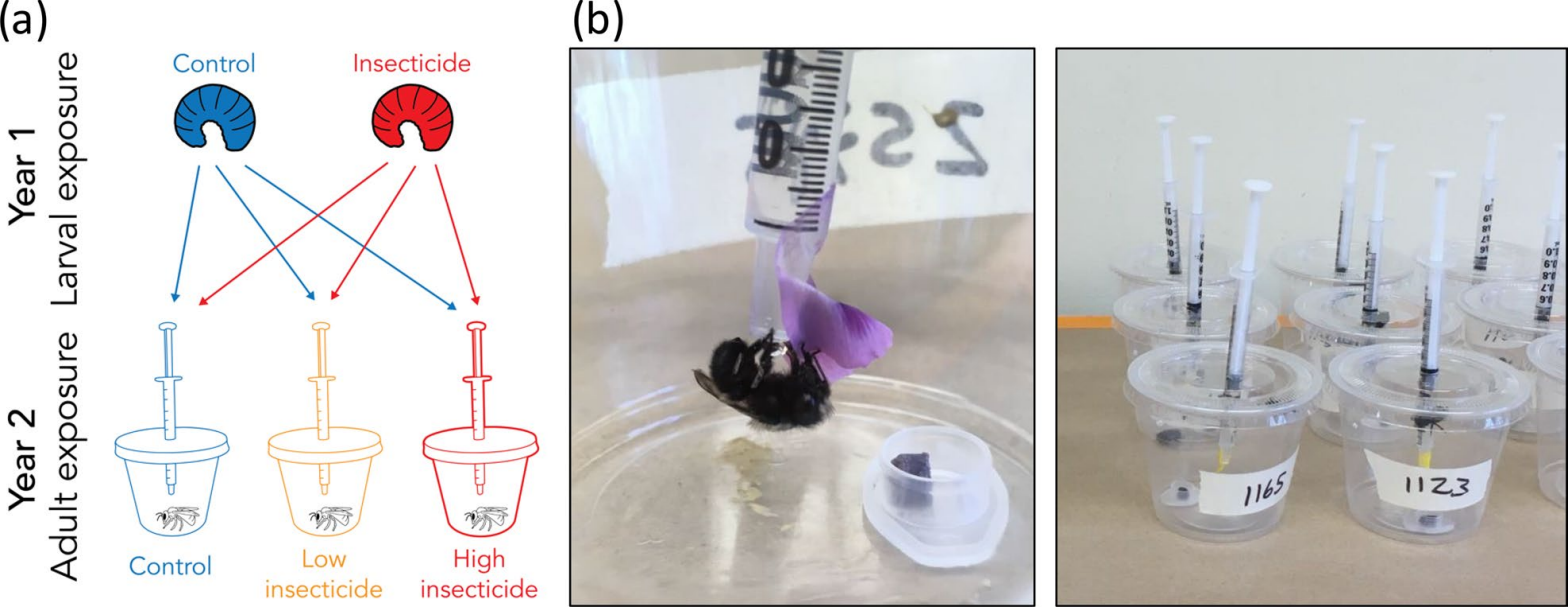
Experimental design. (**a**) Female *Osmia lignaria* with a history of larval insecticide exposure or no larval exposure were placed into cups receiving pollen and nectar with no insecticide exposure (controls), low insecticide exposure, or high insecticide exposure; (**b**) Photos of experimental setup.

For this experiment, I established three insecticide exposure treatments for the adult bees (control, low, and high imidacloprid exposure; see section below for details). Bees from each past treatment group (past larval exposure vs. no past larval exposure to imidacloprid) were crossed with current imidacloprid treatments (no adult exposure, low adult exposure, high adult exposure) in a reciprocal transplant to enable differentiation of the effects of adult exposure vs. those due to past larval exposure (Fig. 1a).

### Adult experimental setup

I incubated cocoons containing adult female *O. lignaria* at 24 °C until bees emerged. Upon emergence, I placed individual bees into clear plastic deli cups (height: 6 cm, diameter: 4.75–7.25 cm) with lids and air holes. Each cup contained an artificial nectar feeder and a pollen feeder (see diet preparation details below). Nectar was provided *ad libitum* via a 1 mL syringe. I attached a flower petal to the tip of each syringe to assist bees in locating and accessing the feeder (Sgolastra et al. 2018; Azpiazu et al. 2019). Each bee also received a 55 mg pollen provision in a microcentrifuge tube lid at the bottom of each cup. Bees were kept in a climate-controlled laboratory at 24 °C in natural light conditions (Fig. 1b).

*Osmia* ovaries typically develop within 3–4 days of emergence (Sgolastra et al. 2010; Lee et al. 2015). Therefore, I maintained bees in the cups for a 4-day feeding and exposure period to coincide with this ovary maturation window. Bees had continuous access to their assigned nectar and pollen treatments throughout the full 4 days. Bees that died before the end of the 4-day trial were excluded from analysis.

### Adult insecticide treatments

The neonicotinoid insecticide imidacloprid was used for the insecticide exposures in this study. Imidacloprid is widely applied across the United States and worldwide (Jeschke et al. 2011; Bass et al. 2015; California Department of Pesticide Regulation 2023). As a systemic insecticide, imidacloprid is taken up by plants and distributed throughout all tissues, including pollen and nectar, posing a significant risk to bees (Jeschke et al. 2011; Goulson 2013).

Adult bees were randomly assigned to one of three exposure treatments: control (no exposure), low (0.7 ppb imidacloprid in nectar, 6 ppb in pollen), or high (10 ppb in nectar, 12 ppb in pollen). These concentrations are within the field-realistic range of imidacloprid concentration in pollen and nectar (Bonmatin et al. 2005; Mullin et al. 2010; Cresswell 2011; Blacquière et al. 2012; Rundlöf et al. 2022) and have been replicated in other bee exposure studies (Whitehorn et al. 2012; Gill et al. 2012; Feltham et al. 2014). These exposure levels are conservative compared to a similar study on *Osmia* ovary maturation (Azpiazu et al. 2019) and other estimates of imidacloprid in bee-collected pollen (Mullin et al. 2010; Blacquière et al. 2012; Rundlöf et al. 2022).

### Diet preparation

Artificial nectar consisted of a 50% sugar solution in distilled water with a sorbic acid solution added at a rate of 5 mL/L to prevent mold growth (Rowe et al. 2023). I obtained pollen from fresh provisions collected by *O. lignaria* foraging on *Phacelia tanacetifolia* flowers in an outdoor flight cage, which had not been treated with pesticides (maintained for over 10 years with no inputs at the University of California, Davis Bee Biology Facility). I homogenized the collected provisions and mixed in a small amount of distilled water to achieve the correct consistency (Sgolastra et al. 2018).

To produce the imidacloprid-treated diets, I prepared a stock solution of 100 ppm imidacloprid by diluting Admire^®^Pro (42.8% imidacloprid; Bayer Crop Science) into distilled water. I diluted the stock solution to the appropriate concentrations for study treatments (see adult insecticide treatments, above), which I then added directly to the nectar or distilled water for the pollen. Control nectar and pollen received untreated distilled water instead of the imidacloprid solution.

### Feeding measurements and ovary dissections

After the 4-day exposure period, I froze bees at -20 °C for later dissection and measured pollen and nectar consumption for each bee. I also set up ten additional cups without bees to control for evaporation from the nectar and pollen feeders and corrected the consumption measurements for evaporation.

To assess ovary development, I dissected bees in phosphate-buffered saline and measured the length of each terminal oocyte. I also measured the head width of each bee using digital calipers (Bosch and Vicens 2002), as head width is a reliable proxy for overall body size in *O. lignaria* (McCabe et al. 2021). In total, I measured ovaries for 183 bees (sample sizes by treatment provided in Table S1).

### Statistical analysis

I used a linear model framework to analyze the effects of imidacloprid exposure on *O. lignaria* nectar consumption, pollen consumption, and ovary development (length of the longest oocyte) (Table S2) using the R package ‘glmmTMB’ (Brooks et al. 2017). For all models, I included adult insecticide exposure (control, low, high), larval insecticide exposure (exposure, no exposure), and bee body size (head width) as fixed effects. Adult body size is fixed at emergence, so only past larval exposure could influence size; this effect was previously quantified in this cohort and found to be small (Stuligross and Williams 2020). Body size was included as a covariate to account for individual variation and because it can influence feeding and ovary development. To evaluate the impacts of insecticide exposure on ovary development, I used two complementary modeling approaches: one that excluded feeding variables and one that incorporated (1) nectar consumption and (2) pollen consumption as covariates. Nectar and pollen consumption were included in separate models due to their correlation.

Insecticide exposure did not interact between larval and adult exposure for any response variable (Table S3), so I removed the interaction term from final models. The response variables of ovary development, nectar consumption, and pollen consumption were log-transformed to meet normality and homogeneity of variance assumptions. I graphically assessed requirements of distribution and variance homogeneity for all models. P-values were calculated using likelihood ratio tests. Post-hoc Tukey’s HSD tests were calculated using ‘emmeans’ (Lenth 2024). See Table S2 for a list of all tested linear models and results.

To further examine the relationship between insecticide exposure, nectar consumption, body size, and ovary development, I fit a structural equation model using the R package ‘lavaan’ (Rosseel 2012). See Table S4 for the full model structure and results. I conducted all analyses in R version 4.3.2 (R Core Team 2023).

## Results

### Nectar and pollen consumption

**Fig. 2:**
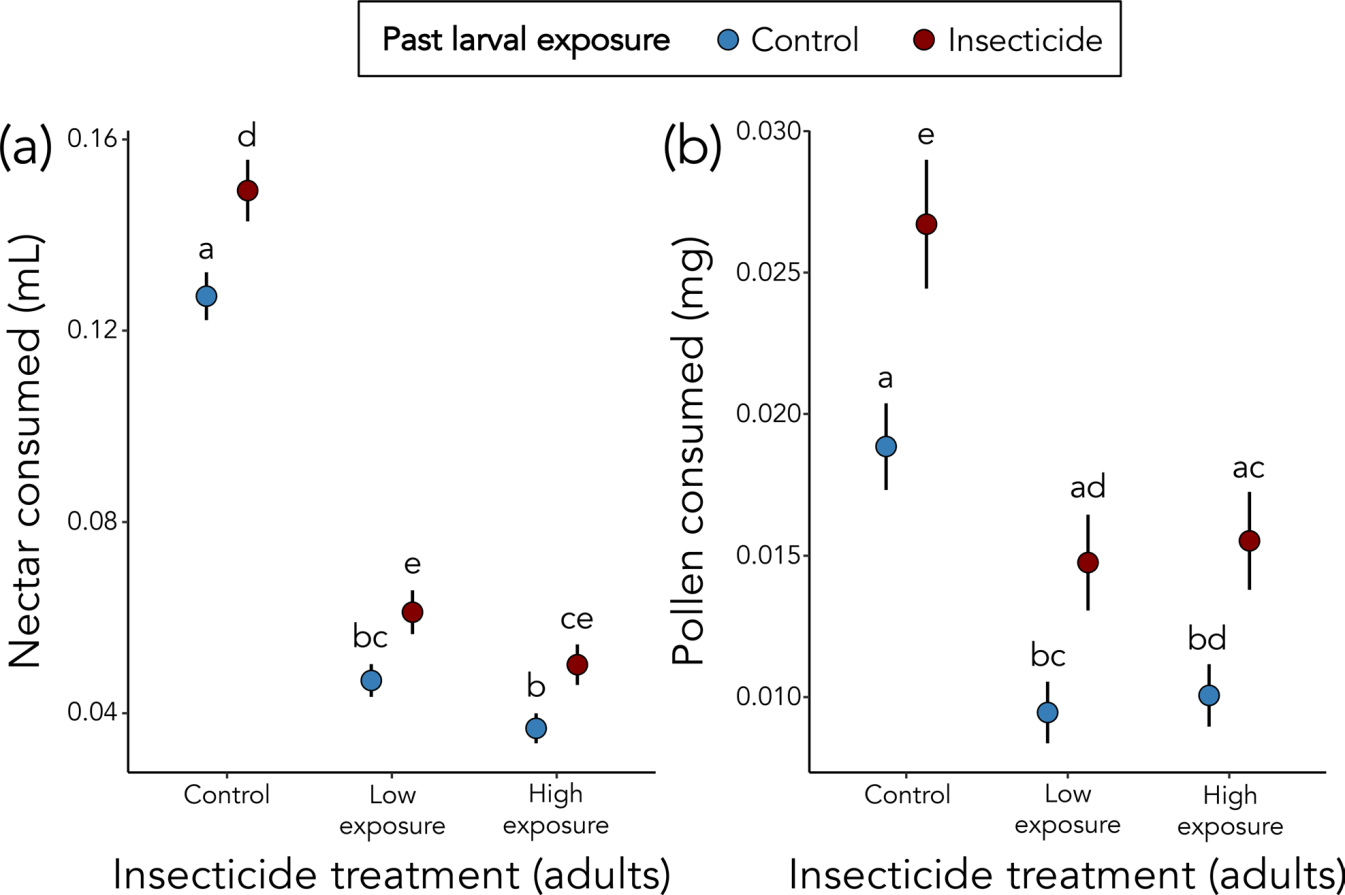
Effects of insecticide exposure on female *Osmia lignaria* food consumption. (**a**) Nectar consumption and (**b**) pollen consumption over four days for adult bees exposed to insecticide as adults and/or as larvae (red: larval exposure, blue: no larval exposure). Error bars are SEs. Letters indicate results of post-hoc Tukey tests, with different letters indicating significant pairwise differences (*p* < 0.05)

*Osmia lignaria* exposed to insecticides as adults consumed less nectar (156% decrease for low exposure and 218% decrease for high exposure; χ^2^ = 201.65, *p* < 0.001) and pollen (89% decrease for low exposure and 79% decrease for high exposure; χ^2^ = 36.58, *p* < 0.001) than unexposed adults (Fig. 2). In contrast, female bees with a history of larval insecticide exposure consumed significantly more nectar (24% increase; χ^2^ = 11.35, *p* < 0.001) and pollen (49% increase; χ^2^ = 13.19, *p* < 0.001) than bees that were not exposed as larvae (Fig. 2). Nectar and pollen consumption did not significantly differ between the low and high insecticide treatments (nectar: *p* = 0.190; pollen: *p* = 0.998; Fig. 2). Body size did not significantly influence nectar (χ^2^ = 1.58, *p* = 0.208) or pollen consumption (χ^2^ = 2.56, *p* = 0.110).

### Ovary development

**Fig. 3:**
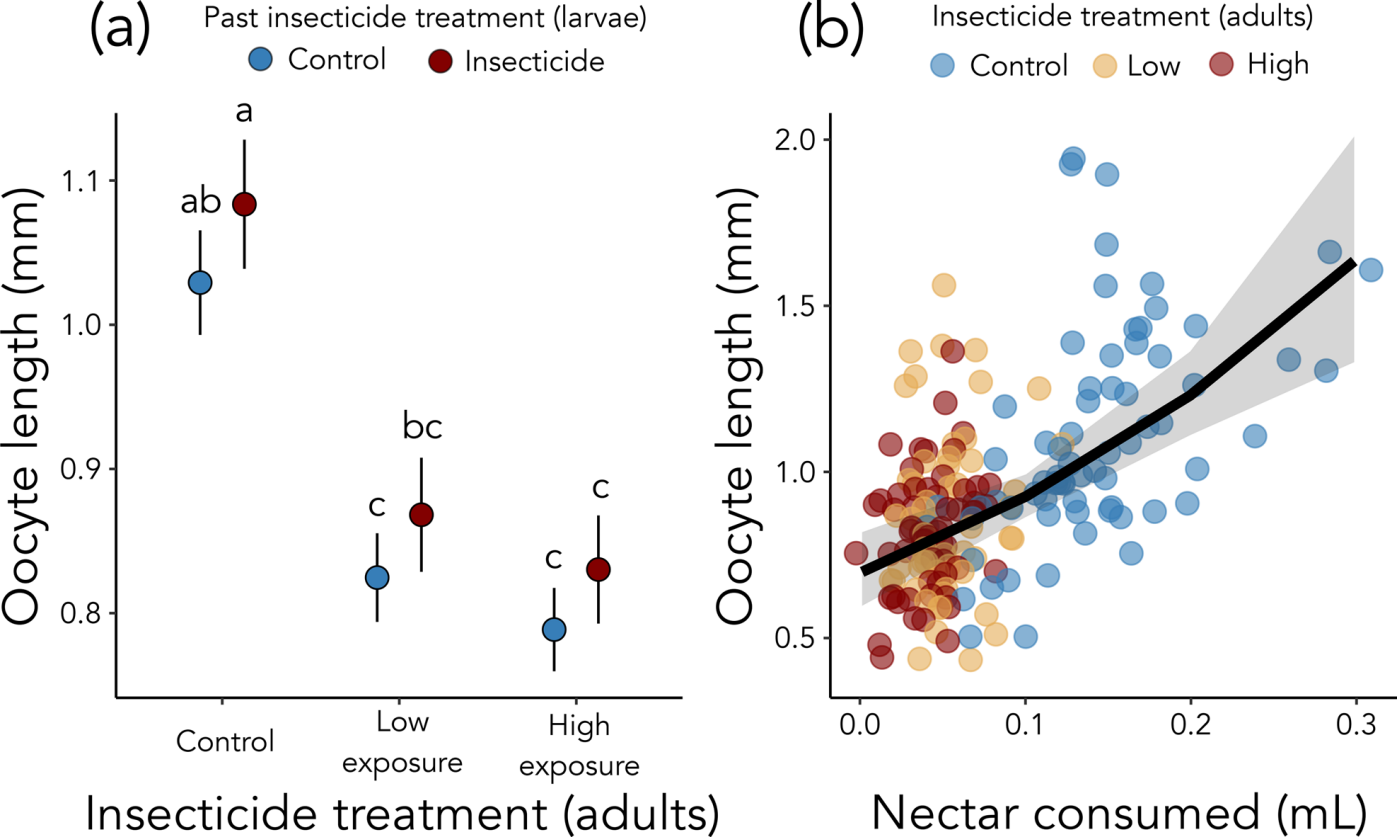
Effects of insecticide exposure and nectar feeding on ovary development. (**a**) Length of adult female *Osmia lignaria* basal oocyte four days post-emergence for bees exposed to insecticide as adults and/or as larvae (red: larval exposure; blue: no larval exposure). Letters indicate results of post-hoc Tukey tests (*p* < 0.05). (**b**) Oocyte length for bees in relation to the amount of nectar consumed. Adult insecticide exposure is indicated by color (blue: control; yellow: low exposure; red: high exposure)

To distinguish direct effects of insecticide exposure on ovary development from those mediated through feeding, I report results from two complementary modeling approaches. I first examined treatment effects on oocyte length, then evaluated whether these effects were explained by variation in nectar and pollen consumption. The first approach revealed that imidacloprid exposure by adult *O. lignaria* impaired ovary development (25% reduction in oocyte length for low exposure and 30% reduction for high exposure; χ^2^ = 35.18, *p* < 0.001; Fig. 3a). However, past insecticide exposure to *O. lignaria* larvae did not influence adult oocyte length (χ^2^ = 1.40, *p* = 0.236; Fig. 3a). Oocyte length did not significantly differ between the low and high insecticide treatments (*p* = 0.941; Fig. 3a) and was positively related to body size across all treatments (χ^2^ = 11.32, *p* < 0.001; Fig. S1).

**Fig. 4:**
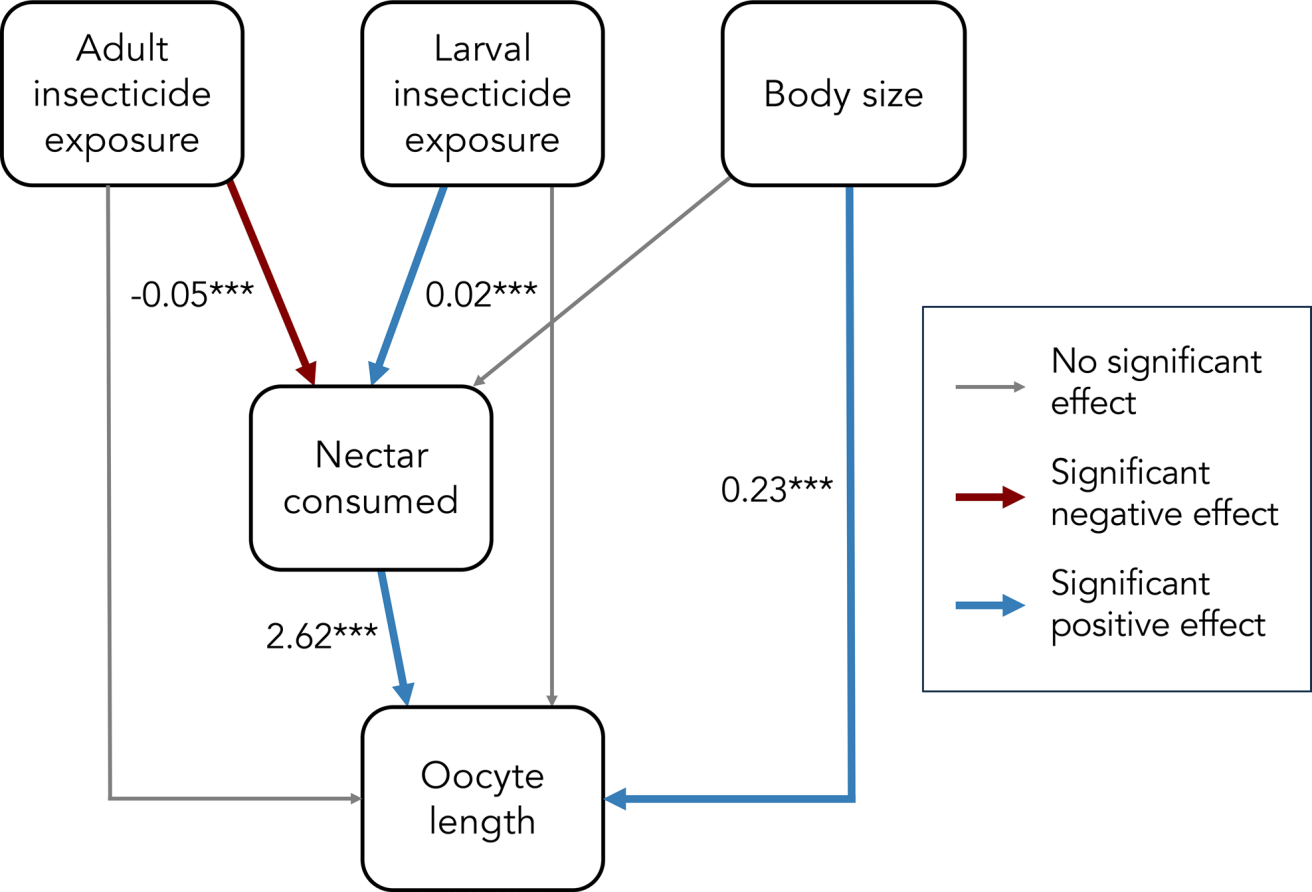
Path analysis for the relationships between insecticide exposure, body size, nectar consumption, and oocyte length. Blue arrows indicate a significant, positive effect; red arrows indicate a significant, negative effect; and gray arrows indicate no significant effect. Numbers adjacent to the arrows represent the estimated strength of the relationship (path coefficients), and stars indicate statistical significance (*** = *p* < 0.001)

Interestingly, the second modeling approach indicated that the ovary impairment from imidacloprid appeared to be strongly driven by a reduction in nectar feeding (Figs. 3b and 4). When nectar and pollen consumption were included as covariates, female oocyte length significantly increased with nectar consumption (χ^2^ = 23.62, *p* < 0.001; Fig. 3b) but was not related to pollen consumption (χ^2^ = 1.82, *p* = 0.177). With nectar consumption as a covariate, imidacloprid exposure did not directly influence oocyte length (exposure to adults: χ^2^ = 0.143, *p* = 0.931; past exposure to larvae: χ^2^ = 0.0002, *p* = 0.989). A structural equation model further supported that imidacloprid exposure by adult *O. lignaria* impaired ovary development predominantly through a reduction in feeding (Fig. 4, Table S4).

## Discussion

Pesticide risks to bees, driven largely by highly toxic insecticides like neonicotinoids (Douglas et al. 2020; Schulz et al. 2021), threaten bee populations and pollination services through reductions in foraging and reproduction (Rundlöf et al. 2015; Siviter et al. 2021; Stuligross and Williams 2021). However, we still do not fully understand the mechanisms underlying the impacts of neonicotinoids on bee reproduction. Recent research shows delays in nesting initiation and egg-laying in exposed bees (Wu-Smart and Spivak 2018; Leza et al. 2018; Stuligross and Williams 2020, 2021) and points towards impacts on early reproductive behavior and physiological processes, such as ovary maturation. The effects of neonicotinoids also differ based on the timing of exposure, and it is clear that exposure during early life stages can carry over to affect adult reproduction (Stuligross and Williams 2021). By investigating the impacts of imidacloprid at multiple life stages over two years, this study reveals that neonicotinoid exposure of females as adults, but not as larvae, dramatically reduced nectar and pollen feeding, with subsequent impairment of ovary maturation (Figs. 2 and 3).

Adult bees exposed to imidacloprid consumed less nectar and pollen than unexposed adults. Imidacloprid has been documented to suppress feeding in many insects, including *Osmia bicornis* (adults: (Azpiazu et al. 2019), larvae: (Claus et al. 2021)) and *Bombus* (Laycock et al. 2012; Muth et al. 2020; Paus-Knudsen et al. 2023). Bees cannot detect imidacloprid in nectar (Kessler et al. 2015; Muth et al. 2020), but exposure reduces feeding motivation and foraging activity (Gill and Raine 2014; Lämsä et al. 2018; Muth et al. 2020; Stuligross et al. 2023). It is possible that bees could learn to avoid toxic nectar after experiencing negative effects (Wright et al. 2010), but this has not been demonstrated for imidacloprid (Muth et al. 2020). Thus, it is unclear whether this feeding suppression is due to intentional avoidance of the exposure source or a physiological or neurological impairment of feeding ability, as imidacloprid acts on nicotinic acetylcholine receptors that can alter feeding motivation and motor function (Blacquière et al. 2012). Because feeding was measured only during the exposure period, this study cannot distinguish between these mechanisms; offering insecticide-free diets after exposure would help disentangle these possibilities in future work.

In contrast, bees with past exposure to imidacloprid as larvae exhibited the opposite trend—consuming more pollen and nectar than individuals with no past exposure, even when they were not subsequently exposed as adults (Fig. 2). This carryover effect indicates that imidacloprid exposure at an earlier life stage can alter bees’ feeding behavior as adults. Several mechanisms may underlie this pattern. One possibility is a compensatory response, in which adults increase food intake to offset nutritional deficits or physiological costs incurred during development, such as repairing sublethal damage or meeting elevated metabolic demands associated with detoxification (du Rand et al. 2015). Increased feeding could also support early reproductive development, as diet influences oocyte maturation in *Osmia* (Cane 2016), consistent with the strong relationship between food consumption and oocyte size documented in this study (Figs. 3 and 4). At the same time, increased feeding may carry ecological costs, such as reducing time available for other behaviors (e.g., foraging and nesting). Additionally, in some contexts, increased feeding could increase pesticide consumption and thus exposure to bees, exacerbating impacts of exposure to individuals in intensively managed landscapes.

Another possibility is that increased feeding may be a hormetic response, in which exposure to lower or more intermediate levels of insecticides can have stimulatory effects, sometimes resulting in benefits such as increased activity or growth (Cutler 2013; Cutler and Rix 2015). Hormetic carryover effects have been observed in some other insects (Cutler 2013; Rix et al. 2016; Tang et al. 2019); for example, aphids exposed to imidacloprid three generations prior survived longer under food and water stress than unexposed aphids, but this benefit did not persist when they were exposed to a subsequent insecticide stressor (Rix et al. 2016). Hormesis has not been studied in bees with respect to carryover effects on feeding response, but evidence from prior research in the same study system suggests no benefits from repeated imidacloprid exposure (Stuligross and Williams 2021). Regardless, the negative effects of adult exposure were far greater than the positive effects of past exposure on nectar and pollen feeding, indicating that any potential positive impacts were not sufficient to overcome additional exposure. Further research on this trend would be useful to understand whether this increased feeding persists or whether it is just exhibited in the first days of adult life.

Imidacloprid exposure by adult *O. lignaria* reduced oocyte size, likely driven by a reduction in nectar feeding (Figs. 3 and 4). Some studies on *Osmia* (Sgolastra et al. 2018) and *Bombus* (Baron et al. 2017) have similarly found negative impacts of insecticides on ovary development. On the other hand, many studies have found no relationship or mixed results (Williams et al. 2015; Azpiazu et al. 2019; Siviter et al. 2020). For example, a similar study on *O. bicornis* found that imidacloprid exposure did not influence oocyte length, despite the dramatically higher concentrations of imidacloprid used (Azpiazu et al. 2019). This indicates that *O. bicornis* may be more resilient to imidacloprid exposure than *O. lignaria*. The two species have similar body sizes (*O. bicornis* head width 3.84 mm (Sgolastra et al. 2018) vs. 3.96 mm in this study) but different contact LD50 values for imidacloprid, with *O. bicornis* exhibiting a higher tolerance, although they were not tested in the same study (*O. lignaria*: 0.026 ug/bee (Peterson et al. 2021); O. *bicornis*: 0.03 ug/bee (Uhl et al. 2019) and 0.046 ug/bee (Beadle et al. 2019)). Species-specific differences in neonicotinoid impacts have been documented for *Bombus* (Baron et al. 2017), and oral toxicity values could further explain this difference, as these studies measured exposure through nectar and pollen.

The suppressive effect of imidacloprid on adult feeding appeared to drive the reduction in oocyte size for exposed bees. Pollen consumption by adult *Osmia* is critical for ovary maturation (Cane 2016). Although this study did not find a significant, direct relationship between pollen feeding and ovary maturation, exposed adults consumed only about half as much pollen as unexposed adults (Fig. 2), which may have been insufficient to mature their first oocytes. Additionally, nutritional stress from reduced nectar feeding could have further impaired bees’ ability to tolerate insecticide exposure. Indeed, nutritional stress can interact with insecticides to impair bee health and performance (Tosi et al. 2017; Stuligross and Williams 2020; Knauer et al. 2022), and increased feeding can boost pesticide tolerance (Schmehl et al. 2014).

This study reveals that neonicotinoid exposure to adult bees reduced nectar and pollen feeding, with subsequent reductions in ovary development. Furthermore, imidacloprid exposure to larvae carried over to increase adult feeding behavior in the following year, but it did not have long-term effects on ovary development. These results inform our mechanistic understanding of the impacts of neonicotinoid exposure on bees, suggesting that nesting and egg-laying delays may be attributed to reduced oocyte maturation due to the anti-feeding effects of imidacloprid and potentially modest direct effects of neonicotinoids themselves. These negative impacts of imidacloprid on feeding and ovary development occurred at low doses, indicating that exposure likely impairs wild and managed bee populations. Field studies regularly measure imidacloprid in nectar and pollen at both exposure levels from these trials (Mullin et al. 2010; Blacquière et al. 2012). Agricultural landscapes pose particularly high risks. For example, *O. lignaria* is managed for pollination services in California almond orchards, where pollen collected from nests contained imidacloprid at levels exceeding the high-exposure level from this study (Rundlöf et al. 2022). Reduced foraging motivation from insecticide exposure could impair critical pollination services to these crops (Stanley et al. 2015; Herbertsson et al. 2022), and reduced ovary maturation could limit the long-term population persistence of this species, particularly in agricultural landscapes (Stuligross and Williams 2021; Rundlöf et al. 2022). To fully understand and mitigate the consequences of insecticide exposure, future research should examine effects across all life stages—from early larval development through adulthood—focusing on cumulative and interactive impacts on survival, reproduction, and population dynamics in both managed and wild bee populations.

## Supplementary Information

Below is the link to the electronic supplementary material.

Supplementary Material 1

## Data Availability

The datasets generated and analyzed during this study are available from the Dryad Digital Repository: https://doi.org/10.5061/dryad.vx0k6dk34.
