# Supplementary material for: Insecticide exposure alters feeding and impairs ovary development in a solitary bee
Source: Ecotoxicology. 2026 Feb 18;35(3):45. doi: 10.1007/s10646-025-03019-y (PMC12916508; doi:10.1007/s10646-025-03019-y)
Supplement: Supplementary file 1 — Supplementary Material 1 [file 10646_2025_3019_MOESM1_ESM.pdf]

Supplementary Information—Insecticide exposure alters feeding and impairs ovary development in a solitary bee. Clara Stuligross

Table S1. Sample sizes.

**Adult insecticide treatment**	**Larval insecticide treatment**	**Number of bees**
Control	Control	42
Control	Pesticide	27
High exposure	Control	40
High exposure	Pesticide	18
Low exposure	Control	39
Low exposure	Pesticide	17

Table S2. Summary results for linear models describing (a) nectar consumption, (b) pollen consumption, and (c – e) ovary development. Chi-sq and p-values are from likelihood ratio tests on nested models with and without the term included.

**Model term**	**Chi-sq**	**p-value**
(a) Nectar consumption		
Adult insecticide treatment	201.65	<0.001
Larval insecticide treatment	11.35	<0.001
Body size	1.16	0.208
(b) Pollen consumption		
Adult insecticide treatment	36.58	<0.001
Larval insecticide treatment	13.19	<0.001
Body size	2.56	0.110
(c) Ovary development		
Adult insecticide treatment	35.18	<0.001
Larval insecticide treatment	1.40	0.236
Body size	11.32	<0.001
(d) Ovary development (including nectar)		
Adult insecticide treatment	0.143	0.931
Larval insecticide treatment	0.0002	0.989
Body size	10.18	0.001
Nectar consumption	23.62	<0.001
(e) Ovary development (including pollen)		
Adult insecticide treatment	22.27	<0.001
Larval insecticide treatment	0.634	0.426
Body size	12.28	<0.001
Pollen consumption	1.82	0.177

Table S3. Results of linear models testing the interactions between larval and adult insecticide exposure on (a) nectar consumption, (b) pollen consumption, and (c – e) ovary development; model letters correspond to model structures from Table S2. Significance was estimated using a likelihood ratio test on nested models with and without the term included.

**Model**	**χ^2**	**P-value**
(a) Nectar consumption	2.44	0.295
(b) Pollen consumption	2.63	0.268
(c) Ovary development	2.80	0.247
(d) Ovary development (including nectar)	4.93	0.085
(e) Ovary development (including pollen)	2.88	0.237

Table S4. Summary results for structural equation model.

**Response**	**Predictor**	**Estimate**	**SE**	**Z-value**	**p-value**
Oocyte length	Adult insecticide treatment	-0.01	0.03	0.24	0.81
	Larval insecticide treatment	-0.02	0.04	-0.48	0.63
	Body size	0.23	0.07	3.23	0.001
	Nectar consumption	2.62	0.44	5.91	<0.001
Nectar consumption	Adult insecticide treatment	-0.05	0.00	-14.32	<0.001
	Larval insecticide treatment	0.02	0.01	3.25	0.001
	Body size	0.01	0.01	1.21	0.23

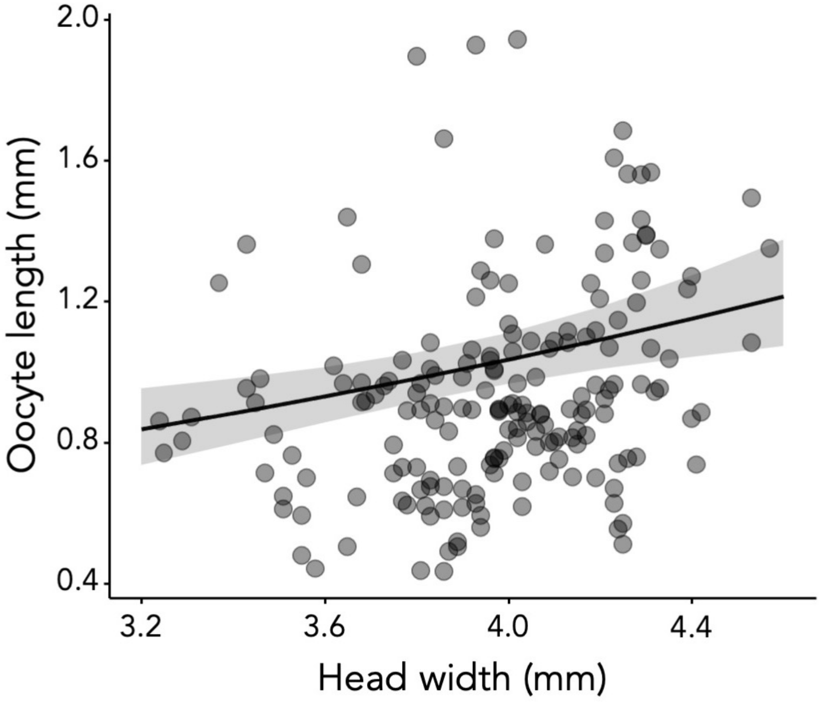

Figure S1. *Osmia lignaria* oocyte length in relation to body size (head width). Shading indicates 95% confidence interval.
